# Diversity of astroviruses in wild animals in Yunnan province, China

**DOI:** 10.1186/s12985-024-02314-0

**Published:** 2024-02-27

**Authors:** Xingyu Huang, Junjie Hou, Xiang Le, Yutong Hou, Lingsi Yang, Qian Li, Binghui Wang, Xueshan Xia

**Affiliations:** Faculty of Life Science and Technology, Kunming University of Science and Technology, 650500 Kunming, Yunnan P.R. China

**Keywords:** Astroviruses, Bastroviruses, Astrovirus diversity

## Abstract

**Background:**

Astroviruses (AstVs) are single-stranded RNA viruses that have been detected in a wide range of mammals and birds. They are associated with numerous interspecies transmissions and viral recombination events, posing a threat to human and animal health.

**Methods:**

We collected 1,333 samples from wild animals, including bats, rodents, wild boars, and birds, from various states and cities in the Yunnan Province, China, between 2020 and 2023 to investigate the presence of AstVs. AstVs were detected using a polymerase chain reaction targeting the *RdRp* gene. Finally, the Molecular Evolutionary Genetics Analysis software was used to construct the phylogenetic tree.

**Results:**

The overall positivity rate for AstVs was 7.12% in four species, indicating their widespread occurrence in the region. High genetic diversity among AstVs was observed in different animal species, suggesting the potential for interspecies transmission, particularly among rodents and birds. Additionally, we identified a novel AstV strain and, for the first time, provided information on the presence of bastroviruses in Yunnan, China.

**Conclusions:**

The widespread distribution and high genetic diversity of AstVs, along with the observed potential for interspecies transmission, highlight the importance of further investigation and surveillance in the region. The findings emphasize the need for increased attention to AstVs and their potential impact on human and animal health in Yunnan and other regions.

**Supplementary Information:**

The online version contains supplementary material available at 10.1186/s12985-024-02314-0.

## Background

Astrovirus (AstV) is a single-stranded positive-sense RNA virus belonging to the *Astroviridae* family. It is a relatively small virus with a genome size of approximately 6.4–7.9 kb [1]. AstVs are classified into two genera: *Mammastrovirus (MAstV)* and *Avastrovirus* (*AAstV)*. The name “Astrovirus” is derived from its distinctive star-shaped structure, resembling a dodecahedron. AstVs contain three essential open reading frames (ORFs): ORF1a encoding non-structural proteins that assist in generating functional proteins during the replication process; ORF1b encoding an RNA-dependent RNA polymerase (RdRp) responsible for replicating the viral genome; and ORF2 encoding the primary structural protein that forms the outer shell of the viral particles. These structural proteins play a crucial role in protecting the genome and interacting with host cells, leading to infection and the onset of symptoms in the host [2]. Human AstV (HAstV) is an important pathogen responsible for diarrhea in infants and young children [3]. It can cause more severe symptoms in individuals with weakened immune systems, such as immunodeficient patients and those undergoing long-term cancer treatment. Additionally, HAstV can infect the human brain, leading to encephalitis and meningitis [4]. Compared to mammals, avian species infected with AstVs tend to experience more severe symptoms. AstVs in poultry, also known as AAstVs, induce poultry enteritis death syndrome, broiler growth retardation syndrome, kidney and visceral gout in broilers, and fatal hepatitis in ducklings [5], causing detrimental effects on the growth and development of poultry, leading to stunted growth, increased mortality, and substantial economic losses. Over the past half century, AAstVs have caused widespread epidemics in various host species [6].

Since their discovery in 1975, AstVs have demonstrated remarkable genetic diversity and a wide host range, attracting significant biological research interest. Previous studies classified AstVs based on their host species of origin. However, our understanding of AstVs has expanded with advancing research, revealing their ability to infect diverse animal populations and prompting further investigation of their biological characteristics and epidemiology. The extensive host range of AstVs is truly impressive, including poultry, livestock (such as pigs, cows, and sheep), fish, reptiles, and birds [7]. The wide host range of AstVs highlights their adaptability and potential for cross-species transmission, necessitating an understanding of their epidemiology and the development of appropriate control measures. Different types of AstVs typically infect specific host populations, and host specificity may vary. Notably, numerous novel AstVs have been identified with the development of sequencing technologies. Classifying AstVs based solely on host species is no longer sufficient to encompass the vast genetic diversity of these viruses. In 2010, the International Committee on Taxonomy of Viruses (ICTV) proposed a classification system for AstVs based on the amino acid sequences of the ORF2 genomic region. According to this system, the genus *MAstV* was classified into *MAstV-1–19*, and *AAstV* was classified into *AAstV-1*–*3* [8]. This classification approach based on the ORF2 gene region allows for a more comprehensive understanding of the genetic diversity and evolutionary relationships among AstVs. To date, a significant number of AstVs remain unclassified, primarily because of the limited availability of ORF2 sequences, which makes consistent classification challenging [7]. Another contributing factor is the high mutation rate of AstVs, which enables recombination events between different strains, resulting in high genetic diversity and facilitating cross-species transmission. Furthermore, the ability of AstVs to undergo recombination events and cross-species transmission highlights their adaptive nature and potential for emergence as novel pathogens, emphasizing the importance of surveillance and research efforts to monitor AstV diversity, understand their ecological dynamics, and identify potential threats to public health.

Recently, a group of hepe-AstV-like RNA viruses associated with AstVs were identified and named bastroviruses (BastVs). BastVs have been found in the feces of healthy humans [9], pigs [10], bats [11], and rodents. Unlike AstVs, BastVs encode only two ORFs. ORF1, which encodes non-structural proteins, shows high homology to the Hepatitis E virus (HEV) and ORF2, which encodes a structural protein, is closely related to the AstVs. Therefore, BastVs are considered recombinant viruses formed through recombination events between AstVs and HEV [9].

In recent years, many new and re-emerging infectious diseases have appeared globally, with increasing occurrences of cross-species transmission of viruses originating from animals [12, 13]. Notably, over half of human diseases are caused by pathogens that originate in animals [14, 15], with bats, rodents, and birds serving as important reservoirs. Therefore, conducting epidemic surveillance of diseases in wildlife is crucial to gain insights into the virus before it becomes highly pathogenic [16]. This proactive approach helps to take preventive measures in advance, and effectively protect the human population. The Yunnan Province is renowned for its rich biodiversity and abundant animal and plant resources. However, the research on AstVs in this region is limited. Considering the prevalence of diseases and the potential for cross-species transmission, studying the epidemiology and evolutionary relationships of AstVs in wild animals is crucial for gaining a better understanding of their genetic diversity and evolutionary dynamics. This knowledge is invaluable for the early detection, rapid response, and implementation of effective control measures to mitigate the impact of emerging infectious diseases. Further, understanding the ecological factors and host–pathogen interactions in wildlife provides important insights into the evolution and spread of infectious diseases. In this study, we discovered a novel AstV through comparative genomics and phylogenetic analyses, observed the potential characteristics of interspecies transmission, and, for the first time, reported the presence of BastVs in Yunnan, China.

## Methods

### Animal trapping and sample collection

The samples used in this study were collected from various states and cities in the Yunnan Province, China, between June 2020 and May 2023. The samples included bats, rodents, wild boars, and birds. To collect wild rodents, trapping devices were placed near natural habitats and human settlements in the evening. The traps were retrieved early in the morning the following day to ensure the freshness of the rodent samples. The captured rodents were euthanized and placed on dry ice for transportation while maintaining their integrity. Most wild boar *(Sus scrofa)* samples were collected by trained forest rangers. After capturing the wild boars, swab samples were obtained from their bodies after anesthesia, and in certain cases of wild boar dying, tissue samples were collected. In addition, fresh fecal samples from bats and birds were collected with a 1-m interval between each sample to prevent contamination. All swab and fecal samples were placed in a virus preservation solution (hubei, China) and transported to the laboratory on dry ice. They were stored at -80 °C for subsequent laboratory analysis and research. Meticulous sample collection and preservation procedures were employed to ensure the integrity and quality of the samples and to facilitate accurate laboratory investigations.

### Viral nucleic acid extraction and taxonomic assignments

To obtain the required tissue samples, the captured rodent specimens were dissected, and the respective tissue samples were placed in a virus preservation solution for further experiments. After dissection, the waste was properly disposed of to prevent environmental contamination and human exposure. Suspensions from animal tissues, swabs, and fecal samples were prepared using grinding and centrifugation. RNA and DNA extractions were performed using 200 µL of homogenized solution with the Tianamp Virus RNA Kit and Tianamp Genomic DNA Kit, respectively, following the manufacturer’s instructions. After species identification using the *Cytochrome oxidase I* gene and the *Cytochrome B* gene [17, 18], the extracted nucleic acids were stored at -80 °C for further experiments.

### Detection and next-generation sequencing (NGS) of AstVs

AstVs were detected using polymerase chain reaction (PCR) targeting the *RdRp* gene, using a previously established method [19]. The PCR system and conditions were set according to the manufacturer’s instructions. Subsequently, a representative subset of AstV-positive samples in four species was selected for NGS, which was conducted at Magigene. The sequencing data obtained were subjected to de novo assembly using an automated pipeline. During the assembly process, viral reads were identified using DIAMOND protein alignment and compared with the Swissprot Uniref 90 database. Identified viral reads were grouped based on their lowest common ancestors and assembled using SPAdes. The resulting contigs were further aligned and merged using Advanced Genome Aligner. To validate the obtained sequences and acquire the complete genome, primers were designed using DNASTAR and Oligo 7. These primers were then used to verify and amplify the full-length genome.

### Genomic analysis of the AstVs and BastVs

ORFs were predicted using ORFfinder (NCBI: https://www.ncbi.nlm.nih.gov/orffinder/). cNLS Mapper42 was used for nuclear localization signals, and National Center for Biotechnology Information (NCBI) Conserved Domains (NCBI: https://www.ncbi.nlm.nih.gov/Structure/cdd/wrpsb.cgi) was used to identify serine protease domains, RdRp, and the conserved region of the capsid.

### Phylogenetic and homology analyses

Reference strains of AstVs were downloaded from GenBank. Sequence alignment and editing were performed using ClustalW (version 2.0) and BioEdit (version 7.1.9), respectively. Subsequently, the Molecular Evolutionary Genetics Analysis (MEGA)-X software was used to determine the optimal model for DNA/protein analysis. The best-fit model selected using MEGA-X was used to construct a neighbor-joining phylogenetic tree. Notably, 1,000 replicate trees were constructed to assess the robustness of the inferred phylogeny. Bootstrap values > 70% were used as thresholds to validate the grouping accuracy. Homology analysis was conducted on the selected sequences to determine the degree of similarity.

## Results

### Surveillance and identification of AstVs

**Fig. 1:**
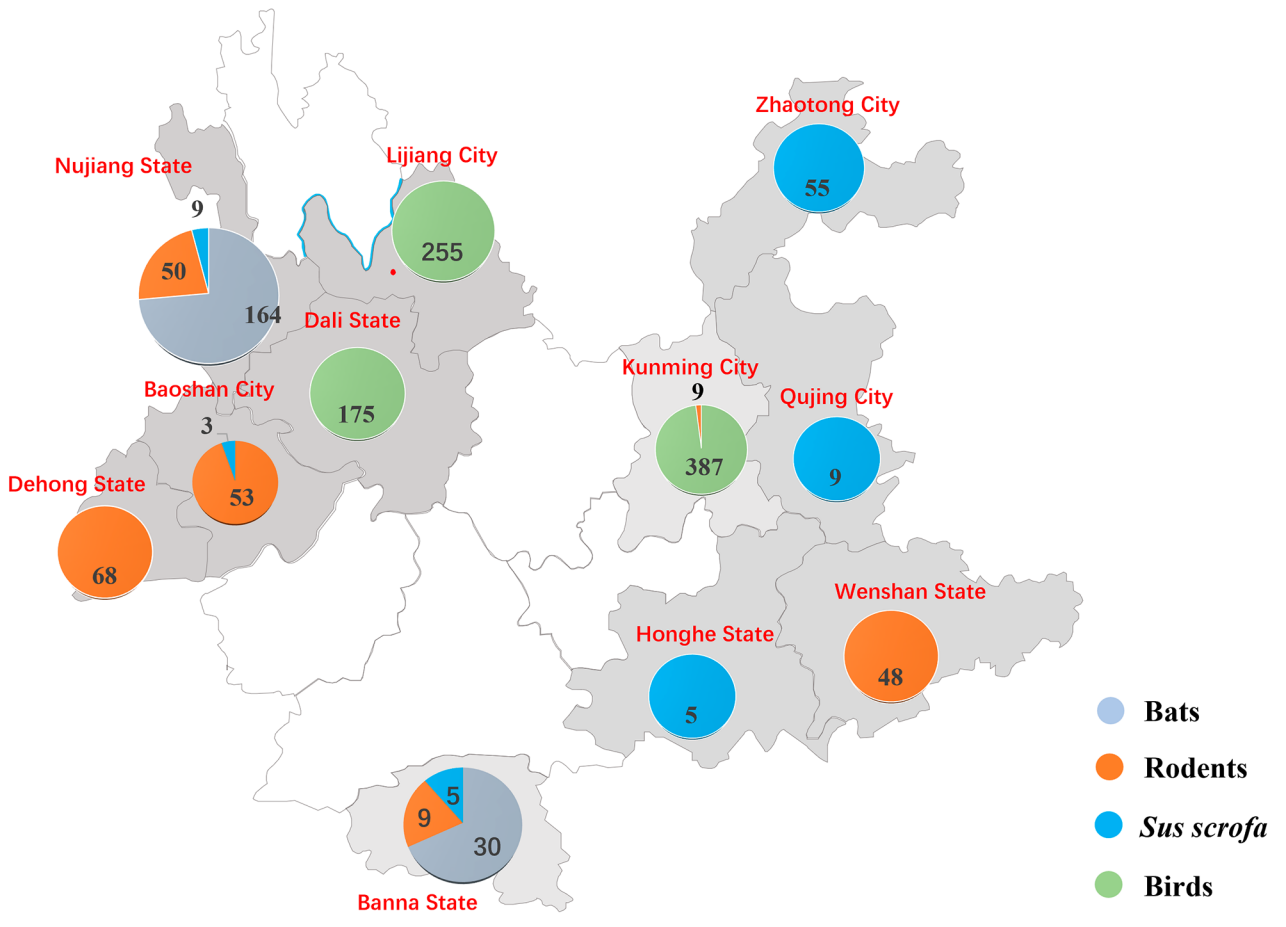
Collection infographic about the samples used in this study

Between June 2020 and May 2023, 1,333 samples of different species were collected from various states/cities in Yunnan, China, including 193 bat, 237 rodent, 86 wild boar, and 817 poultry samples (Fig. 1). Reverse transcription PCR was used to screen for AstVs by targeting their RdRp region. Among the 95 samples (7.12%) that tested positive for AstVs (Table 1), the highest infection rate was observed in wild boars (24.41%), followed by rodents (20.67%), bats (9.32%), and birds (0.50%).

**Table 1:** Samples tested for AstVs in this study

Animal species	No. of Collected Samples	No. of AstVs Positive Samples (%+)
**Bats**		
*Rhinolophus sinicus*	9	0
*Rhinolophus affinis*	10	0
*Hipposideros pomona*	133	13 (9.77%)
*Hipposideros armiger*	11	0
*Miniopterus pusillus*	30	5 (16.66%)
Subtotal	193	18 (9.32%)
**Rodents**		
*Rattus tanezum*	154	37(24.02%)
*Rattus nitidus*	15	1(6.66%)
*Rattus norvegicus*	68	11(16.17%)
Subtotal	237	49(20.67%)
**Suina**		
*Sus scrofa*	86	21(24.41%)
**Birds**		
*Columbiformes*	25	0
*Ciconiiformes*	161	0
*Gruiformes*	67	0
*Charadriiformes*	200	4(2.00%)
*Galliformes*	75	0
*Cuculiformes*	50	1(2.00%)
*Passeriformes*	122	2(1.63%)
*Pelecaniformes*	10	0
*Anseriformes*	107	0
Subtotal	817	7(0.50%)

### Diversity and host range of AstVs

**Fig. 2:**
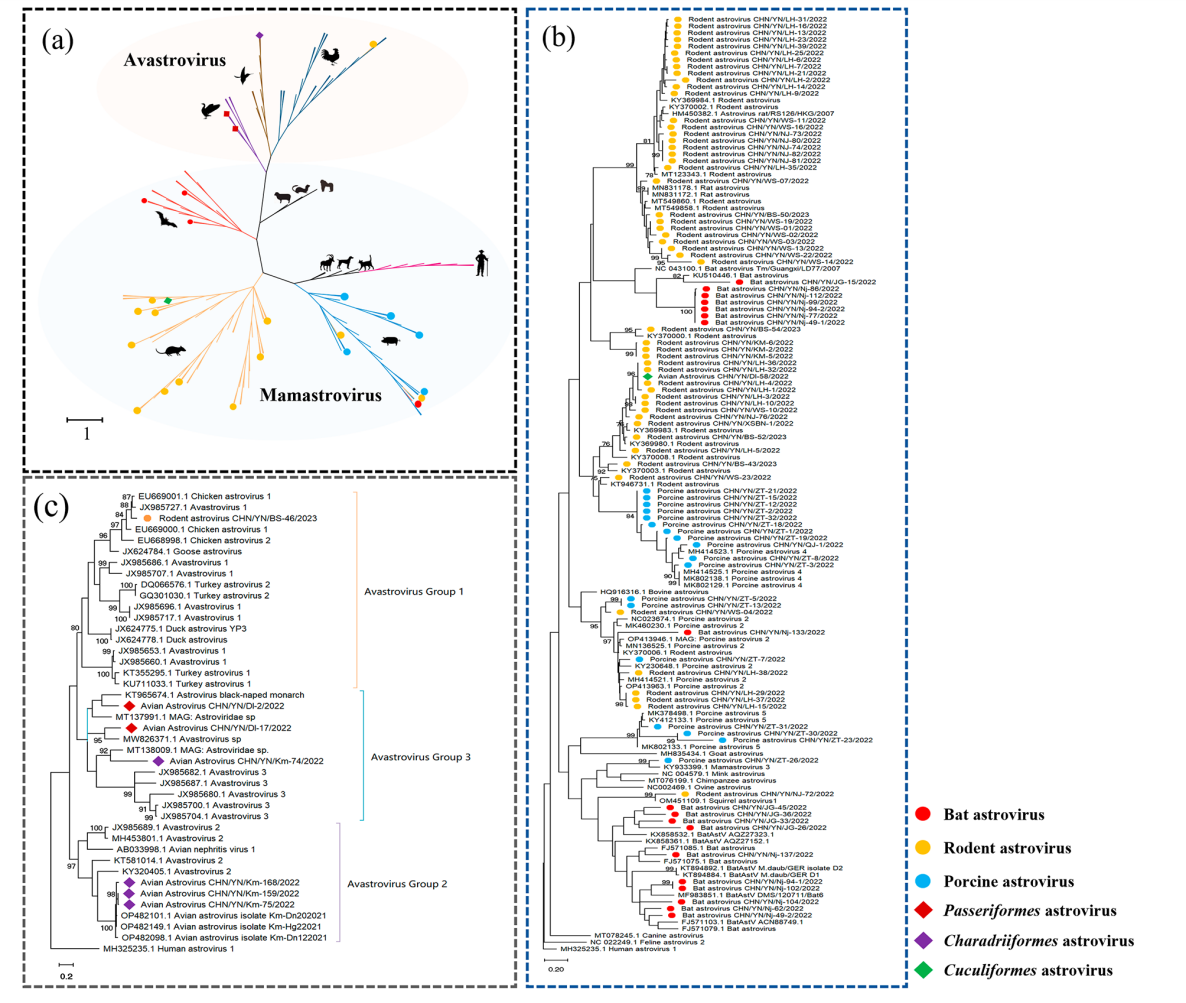
(**a**) According to the partial nucleotide sequences of the RdRp gene (369 bp), sequence comparison and clustering were performed with representative AstVs. Maximum likelihood method was used to construct tree (ML), model (GTR + G), and bootstrap analysis was performed 1000 times; (**b**) phylogenetic tree based on partial nucleotide sequences of RdRp genes (372 bp) of MAstV, model (GTR + G); (**c**) phylogenetic tree based on partial nucleotide sequences of RdRp genes (369 bp) of AAstV, model (GTR + G). The strains obtained in this study are represented by different color symbols

To investigate the genetic characteristics of AstVs, 95 sequences of the *RdRp* gene (422 bp) were obtained and included in the analysis along with 95 reference sequences from the NCBI gene database. As shown in Fig. 2a, the sequences in the phylogenetic tree clustered into two groups: *MAstV* and *AAstV*. Notably, 78 strains from mammals and 1 strain from a bird belonged to the MAstV group. Six strains from birds and one strain from rodents clustered in the AAstV group.

The strains isolated from bats were further divided into two distinct groups. One group exhibited a high degree of diversity and clustered closely with strains of bats from Jiangxi (FJ571079.1) and Cambodia (KX858532.1), whereas the other group formed an independent evolutionary branch and shared high nucleotide homology with bat strains from Gabon (KU510446.1) and Guangxi (NC 043100.1). Multiple AstV strains were detected in the same sample and demonstrated distant evolutionary relationships in the phylogenetic analysis (CHN/YN/Nj-49-1/2022 and CHN/YN/Nj-49-2/2022). Rodent strains formed three main evolutionary branches. The branch represented by CHN/YN/WS-07/2022 was adjacent to the bat-related branch, whereas the branch represented by CHN/YN/WS-23/2022 was adjacent to the monophyletic group porcine AstV (PAstV)-4 and included a strain from *Hierococcyx sparverioides* ( Cuculiformes). The last branch, represented by CHN/YN/KM-5/2022, is evolutionarily distant from the other two branches. The five strains were classified into a monophyletic group formed by PAstV-2. One strain was closely related to the squirrel-associated AstV (OM451109.1). AstVs isolated from wild boar were categorized into three PAstV lineages: PAstV-2, PastV-4, and PastV-5. One strain (BAstV CHN/YN/Nj-133/2022) showed the closest relationship with PAstV-2 (Fig. 2b).

Three groups of AAstVs were observed using phylogenetic analysis of the reference sequences from GenBank and strains isolated from wild birds. Except for the one viral strain described above, six strains, detected from *Larus ridibundus* (Charadriiformes) and *Turdus dissimilis* (Passeriformes), appeared in two groups (Fig. 2c). Avian nephritis virus and its closely related lineages were clustered in group 2. Three AstVs detected in *Larus ridibundus* clustered in this group were closely related to the AAstVs detected in Kunming. Other strains related to strains from several wild duck species reported in Hong Kong were clustered in group. A strain from rodent was grouped into group 1, which included turkey AstV (TAstV)1, TAstV2, duck AstV, duck hepatitis virus 3, and chicken AstV, showing multiple species origins.

### Genomic and phylogenetic analyses of the two rodent AstVs (RoAstVs)

**Fig. 3:**
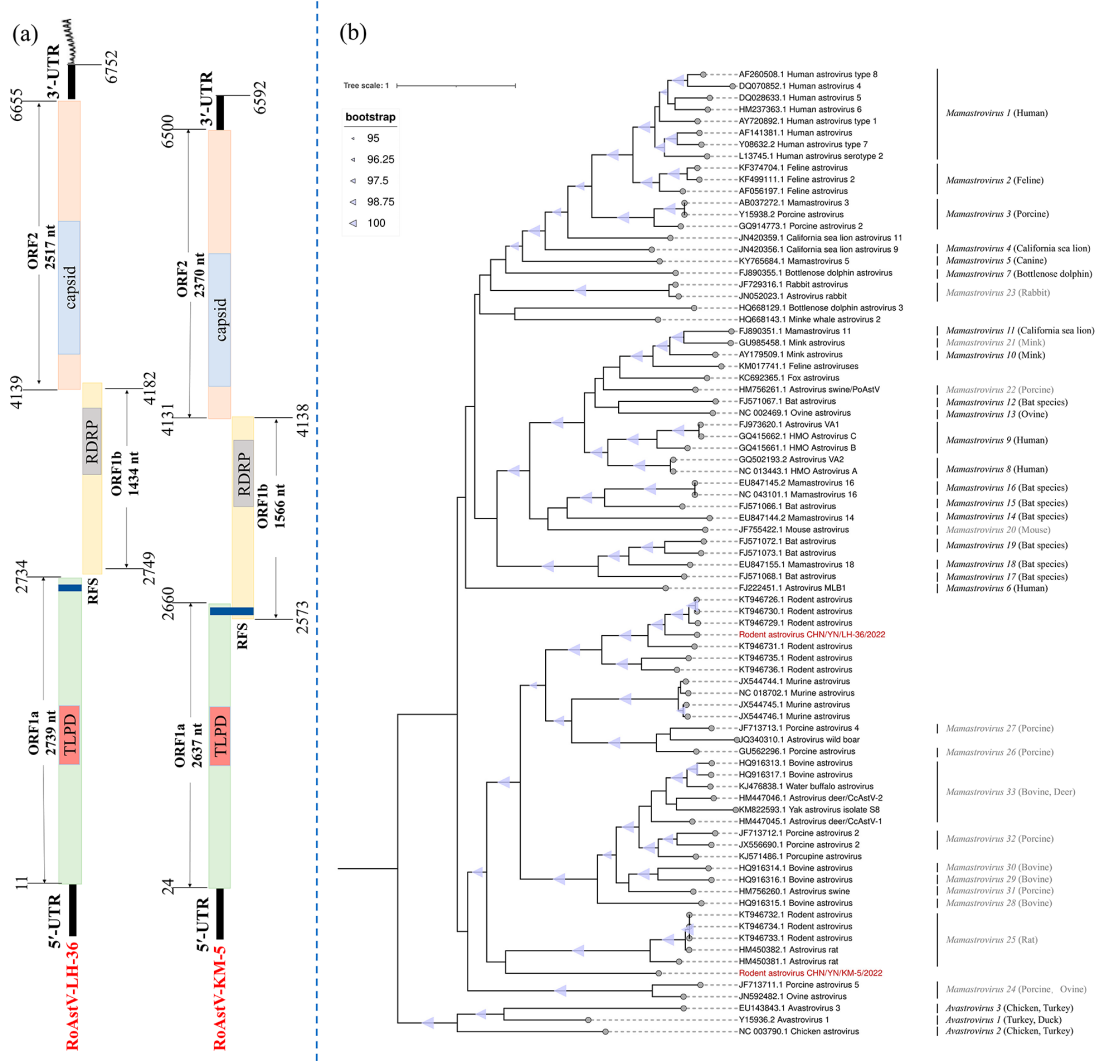
(**a**) Schematic representation of the genomic organization of the two novel RoAstV. TLPD, a Trypsin-like peptidase domain; RFS, a ribosomal frame shift sequence; RdRp, RNA-dependent RNA polymerase; capsid, Astrovirus capsid protein precursor. (**b**) Phylogenetic analysis based on ORF2 sequences of AstVs, Maximum likelihood method was used to construct tree (ML), model (GTR + G + I), and bootstrap analysis was performed 1000 times. RoAstVs characterized in this study are marked in red. Species in black italics are included in the International Committee for Taxonomy of Viruses proposal [20]. Species in grey italics were proposed by Guix et al [21]

In order to acquire more genomic fragments, facilitating full-length genome amplification and enabling a comprehensive exploration of novel AstVs, this study conducted transcriptome sequencing on representative strains from four species representing different evolutionary branches (Table S1). We found the full-length genome data of RoAstVs in pool 15 and pool 14 respectively. Through PCR validation (Table S2), we obtained near-full-length sequences of two RoAstVs (RoAstV-LH-36 [Rodent_astrovirus_CHN/YN/LH-36/2022] and RoAstV-KM-5 [Rodent_astrovirus_CHN/YN/KM-5/2022). Genome ORF prediction revealed that the two strains of RoAstVs exhibited genomic structural features similar to those of most AstVs (Fig. 3a). The strain RoAstV-LH-36 has a ribosomal frameshift sequence (RFS) (nt positions ^2656^AAAAAAC^2662^) located within ORF1a, which is 2,739 nt in length. A trypsin-like peptidase domain (TLPD) is present in the middle of ORF1a (nt positions 1,409–1,768). ORF1b is 1,434 nt in length and contains conserved RdRp motifs at positions 3,181–3,897. The nucleotide sequence (nt positions 4,394–5,335) encoding the capsid protein precursor was located in ORF2 (2,517 nt in length). In the RoAstV-KM-5 strain, RFS (nt positions ^2609^AAAAAAC^2015^) was identified in the overlapping region of ORF1a (2,637 nt in length) and ORF1b (1,566 nt in length). The TLPD is located in the middle region of ORF1a (nt positions 1,305–1,667). ORF1b contains the conserved RdRp sequence at positions 3,134–3,847. The front region of ORF2 (2,370 nt in length) encodes the capsid protein, with the nucleotide sequence ranging from 4,299 to 5,354.

Classic strains of AstVs were selected as reference strains for the phylogenetic analysis of full-length ORF2 (Fig. 3b). Partial reference strains, including the complete ORF2 region, were selected for amino acid homology analysis (Table 2). Phylogenetic analysis showed that the two strains of interest were distantly related. The strain RoAstV-LH-36 clustered with a strain discovered in rodents from Hong Kong (KT946729.1), with a bootstrap value of over 95% and shared 79.59% nucleotide homology. Amino acid homology analysis yielded consistent results. All three ORFs shared the highest homology with strain KT946729.1, with 86.19, 92.20, and 75.48% similarity in ORF1a, ORF1b, and ORF2, respectively. The RoAstV-KM-5 strain was adjacent to but did not cluster with the strain related to MAstV-25, forming a distinct evolutionary branch. Homology analysis revealed that ORF1a and ORF2 had the highest homology with the strain discovered in the Qinghai Province, China (KY855439.1), with 31.94 and 31.31% similarity, respectively. However, ORF1b of this strain exhibited the highest homology (57.93%) with a strain discovered in rodents in Hong Kong (KT946729.1).

**Table 2:** Amino acid homology analysis of two RoAstVs to other AstVs.

Strains	RoAstV-LH-36			RoAstV-KM-5		
	ORF1a(%)	ORF1b(%)	ORF2(%)	ORF1a(%)	ORF1b(%)	ORF2(%)
AY720892.1_Human astrovirus type 1	*22.69* ^a^	56.98	26.24	27.13	56.01	27.40
KT946729.1_Rodent_astrovirus_HK-12,111 F	**86.19**	**92.20**	**75.48**	30.99	**57.93**	28.70
HM450381.1_Astrovirus_rat/RS118/HKG/2007	26.18	55.71	25.01	25.70	57.63	25.89
OM451116.1_Civet_astrovirus _SC-F1	NA^*^	NA	64.24	NA	NA	27.58
OQ198049.1_Canine astrovirus isolate 319 C	NA	NA	42.11	NA	NA	29.21
OL695850.1_Procine astrovirus-4 AH4-3	45.71	70.02	26.96	29.90	56.56	24.39
sKM017741.1_Feline_astrovirus_D1	23.60	*51.70*	*18.72*	*22.06*	*51.44*	22.97
FJ571067.1_Bat_astrovirus Tm/Guangxi/LD71	NA	NA	20.21	NA	NA	*22.48*
KY765684.1_ Fox astrovirus 2016/BRA	23.58	55.29	23.57	25.93	55.71	24.03
KY855439.1_Marmot_astrovirus_3 _HT12	44.84	67.03	43.64	**31.94**	56.82	**31.31**

Highest amino acid identities are in bold and underlined and the lowest identities are italicized. Strains without full-length genome sequences are indicated with a dash (NA) in their corresponding ORF1a and OFR1b.

### Genomic and phylogenetic analyses of rodent BAstVs (RoBAstVs)

**Fig. 4:**
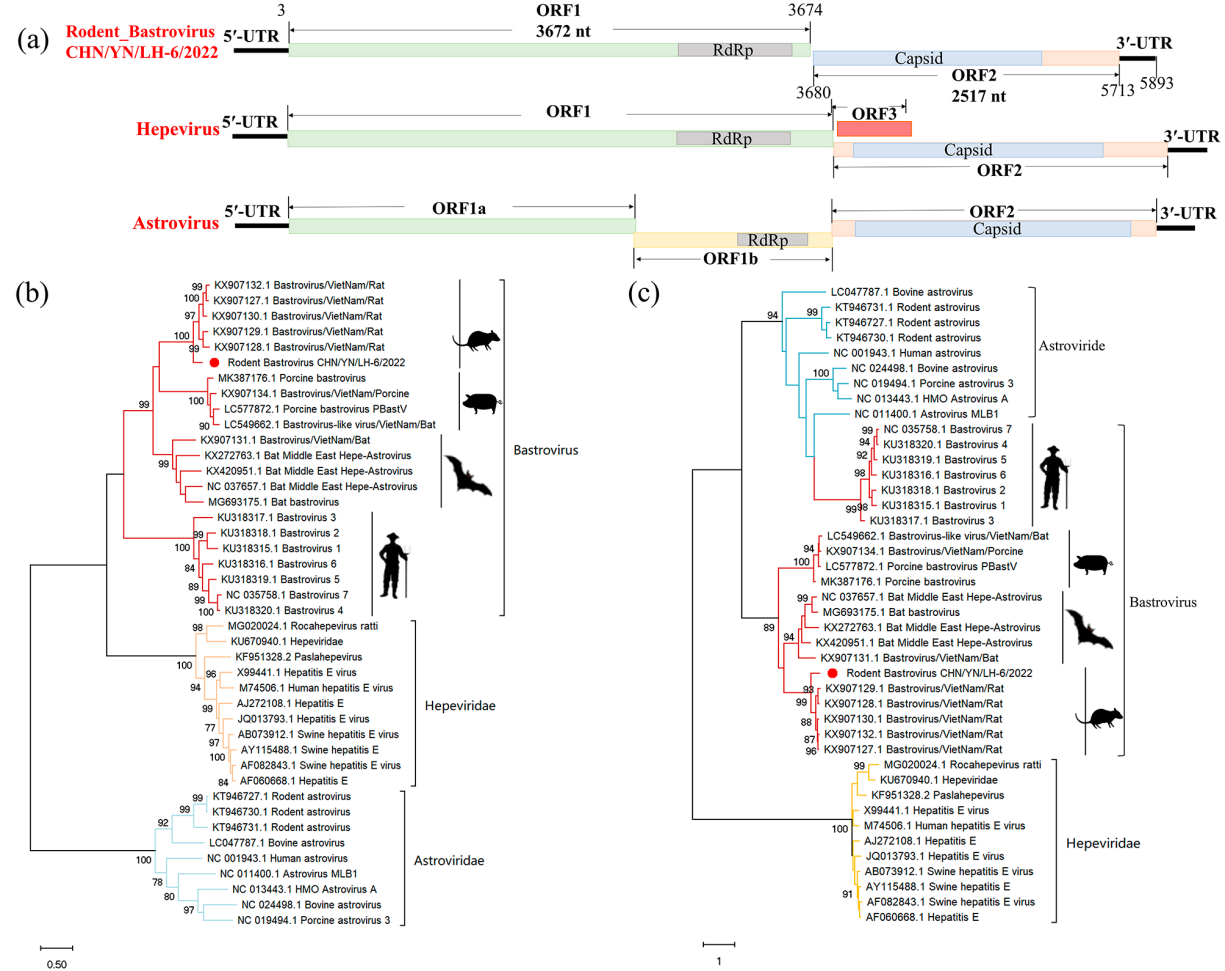
(**a**) Schematic representation of the genomic organization of RoBastv-LH-6. Vmt, viral methyltransferase; Helicase, viral helicase; RdRp, RNA-dependent RNA polymerase; capsid, capsid protein precursor. (**b**) Phylogenetic analysis based on ORF1 sequences of BastVs, HEV and AstVs, Maximum likelihood method was used to construct tree (ML), model (GTR + G + I), and bootstrap analysis was performed 1000 times. (**c**) Phylogenetic analysis based on ORF2 sequences of BastVs, HEV and AstVs, Maximum likelihood method was used to construct tree (ML), model (GTR + G + I), and bootstrap analysis was performed 1000 times. RoBastVs characterized in this study are marked in red

The presence of BAstVs in rodents was discovered In the transcriptome data of pool 18. Based on the transcriptome results, primers were designed to screen for BAstVs in all rodent samples. Three samples were positive for BastV (Table S3). Considering the high nucleotide homology, RoBAstV-LH-6 (Rodent_Bastrovirus_CHN/YN/LH-6/2022) was selected as a representative strain for full-length amplification (Table S4). RoBAstV-LH-6 comprised two ORFs (Fig. 4a). Conserved domains, including viral methyltransferase (nt positions 105–980), viral helicase (nt positions 1,473–2,156), and RdRp (nt positions 2,802–3,278), were distributed within ORF1 (3,672 nt in length), whereas the capsid protein precursor (nt positions 3,644–4,747) spanned across both ORF1 and ORF2 (2,517 nt in length). To understand the phylogenetic relationships of RoBAstV-LH-6, we constructed phylogenetic trees for ORF1 and ORF2 using representative strains from the BastVs and HEV families (Fig. 4b, c). Based on the phylogenetic analysis of ORF1, four well-supported branches were observed, including the Astroviridae family, HEV family, human BastVs, and animal (rodent, bat, and pig)-related BastVs. The evolutionary distance between BastVs and HEV was shorter than that between BastVs and members of the Astroviridae family. In the phylogenetic tree of ORF2, only three highly supported branches were observed. Human BastVs and Astroviridae clustered together, whereas animal BastVs and HEV formed separate clusters. BastVs and the Astroviridae family showed a close evolutionary relationship. Phylogenetic analysis based on different ORFs consistently clustered RoBastVs-LH-6 with rodent-related BastVs, with the highest nucleotide homology of 76.98% (full-length) to the strain from Vietnam (KX907132.1). To explore the amino acid homology of RoBAstV-LH-6, we performed homology analysis using representative strains from the BastV family (Table 3). RoBAstV-LH-6 shared the highest homology with strain KX907132.1, with 89.84 and 72.76% homology to ORF1 and ORF2, respectively.

**Table 3:** Amino acid homology analysis of RoBastv-LH-6 to other BastVs.

Strains	RoBastv-LH-6	
	ORF1a(%)	ORF2(%)
KU318315.1_human_Bastrovirus_1	*26.51*	17.86
KU318316.1_human_Bastrovirus_6	26.65	*17.24*
KX272763.1_Bat_Hepe-Astrovirus KSA239	49.74	33.25
KX420951.1_Bat_Hepe-Astrovirus KSA410	48.12	36.63
KX907129.1_Bastrovirus/VietNam/Rat/16715_10	89.67	72.32
KX907132.1_Bastrovirus/VietNam/Rat/16715_58	**89.84**	**72.76**
KX907134.1_Bastrovirus/VietNam/Porcine/17,489	40.43	25.54
MK387176.1_Porcine bastrovirus USA 2017-1	40.77	25.20

Highest amino acid identities are in bold and underlined and the lowest identities are italicized

## Discussion

AstVs are characterized by their genetic diversity, extensive host range, and cross-species transmission. However, owing to the lack of severe human diseases and large-scale outbreaks associated with AstV infections, they have been neglected by virologists. Currently, most research on AstVs focuses on humans and animals related to livestock farming, whereas little is known about the prevalence and genetic characteristics of AstVs in wild animals. In this study, we aimed to address this knowledge gap by conducting AstV detection in wild animals, such as bats and rodents, which play crucial roles in viral transmission [22–25]. We evaluated the infection rates and genetic features of the viral strains, the detection results revealed that wild boars exhibited the highest AstV infection rate (24.4%), consistent with their known high tolerance to AstV infections [26]. Unlike the significantly high infection rates observed in domestic pigs, the extensive habitat range of wild boars reduces the risk of fecal-oral transmission. The lifestyle habits of rodents, on the other hand, provide ideal opportunities for AstV transmission. In this study, the infection rate among rodents (20.67%) closely resembled previous research findings [27]. Furthermore, varying infection rates were observed among different bat species (ranging from 0 to 16.66%), confirming the species-specific nature of Bat AstV [28]. The infection rate of AstV in wild birds was only 0.50%, which is different from the infection rate in poultry [29]. Moreover, we attempted to provide evidence of interspecies transmission of AstVs among mammals and birds, as well as the phenomenon of co-infection. Through a systematic phylogenetic analysis of 95 AstV strains derived from different animal species, we observed the formation of species-specific and multi-host clusters among these AstVs. RoAstVs typically form multi-host clusters, likely due to the frequent exposure of rodents to the excreta of other animals in their living environment. These findings highlight the strong infectivity and spillover capability of AstVs, revealing their genetic diversity and wide host range [30].

Cross-species transmission is an important way for viruses to break through their inherent hosts and gain more living space, resulting in greater genetic diversity through subsequent adaptation to new hosts. To date, a considerable number of AstV interspecies transmission events have been reported, including cross-species transmission among mammals [31–33], birds [34–36], and between mammals and birds [37]. These transmission events demonstrate that AstVs can infect and rapidly adapt to new hosts due to their high environmental stability [38, 39]. In this study, we discovered that AstV sequences were closely related to PAstVs in bats and rodents. Similar events were observed for AstVs carried by *Hierococcyx sparverioides*, where the viral sequences showed close affinity to AstV sequences from the rodents. Notably, most current research on AstV recombination and transmission is based on similarities with previously published sequences. However, most of these studies rely on partial sequences [27, 40]. The intra- and inter-species transmission could be better understood using the number of complete AstV genomes to truly assess the potential and surveillance of emerging zoonotic AstVs.

Due to the wide host range and high genetic diversity within the AstV family, the ICTV has made several revisions to the classification scheme [41]. In 2010, the ICTV classified AstVs based on the complete amino acid sequence of ORF2, with a minimum homology threshold of 75% for strains of the same species [42]. However, owing to the limited availability of complete genome sequences, a significant number of AstVs remain unclassified. Among the 19 mammalian AstV classifications certified by the ICTV, no strains from rodents have been identified; only two strains, *MAstV-20* (mouse) and *MAstV-25* (rat), have been provisionally classified as new species within the tentative 14 mammalian AstV classifications [43]. Compared to the extensive research on rodents in other viral fields, studies on AstVs in rodents are still insufficient. In this study, we obtained 49 AstV sequences from rodent samples, including two complete viral sequences. The RoAstV-LH-36 strain showed the closest genetic relationship to strain KT946729.1 from Hong Kong, which was classified as a new genotype (Cluster A) in earlier research [44]. Another strain, RoAstV-KM-5, exhibited significant non-homology, with a maximum amino acid homology of only 31.31% in the ORF2. This clearly indicates that it represents a novel AstV species in mammals, which we provisionally named *MAstV-34*. Most rodent samples used in this study were from *Rattus tanezumi* and *R. norvegicus*, which frequently come into close contact with human living environments. Although no cases of AstV infections caused by rodent strains have been reported in humans, the potential interspecies transmission ability of AstVs warrants preventive measures.

BastV was first discovered in human fecal samples in the Netherlands in 2016. Subsequently, it has been identified in bats (Vietnam and Cameroon), rodents, and pigs (the United States and Japan). Due to its unique genomic characteristics, this virus is believed to be a recombinant product of AstV and HEV [45]. In this study, BastVs were found in *R. tanezumi* from Lianghe, which tested positive for AstVs. Owing to high nucleotide homology, we amplified only the full-length sequence of RoBastv-LH-6. From an evolutionary perspective, RoBastv-LH-6 may have been transmitted by Vietnamese rats or shared a common ancestor with them. Nevertheless, RoBastv-LH-6 evolved independently within the rat population in China over a considerable period. Unfortunately, research on BastVs is limited, and fewer than 50 complete BastV nucleotide sequences are available in the NCBI database (BAstV - Nucleotide - NCBI [nih.gov]; accessed on November 18, 2023), therefore, the ICTV has not classified BastVs. Further studies are required to better understand the pathogenicity and genetic characteristics of BastVs.

## Conclusions

AstVs are excellent candidates for studying human, animal, and environmental health. They are highly prevalent worldwide and have been found in a wide range of host species [6]. They pose a significant burden on public health and livestock due to zoonotic transmission to humans and reverse zoonosis to marine mammals [46, 47]. Further research on the epidemiology, pathogenesis, and interspecies transmission mechanisms will contribute to our understanding of the evolutionary history of the viral kingdom and the driving forces behind viral genetic diversity. In this study, we identified many AstV sequences and described their genetic characteristics. Additionally, we identified a novel mammalian AstV strain and, for the first time, provided the evidence of the presence of BastV in Yunnan, China. These findings highlight the need for further research and surveillance to better understand the epidemiology, pathogenicity, and transmission dynamics of AstVs, including newly discovered AstVs, and the prevalence of BastVs in specific regions, such as Yunnan, China. In addition, our findings may help develop the appropriate measures to prevent widespread epidemics caused by AstVs in various host species.

### Electronic supplementary material

Below is the link to the electronic supplementary material.

Supplementary Material 1

Supplementary Material 2

Supplementary Material 3

Supplementary Material 4

Supplementary Material 5

## Data Availability

Data is provided within the supplementary information files.

## Abbreviations

AstVs: Astroviruses
*AAstV*: *Avastrovirus*
BastVs: Bastroviruses
HEV: Hepatitis E virus
HAstV: Human AstV
ICTV: International Committee on Taxonomy of Viruses
*MAstV*: *Mammastrovirus*
NCBI: National Center for Biotechnology Information
NGS: Next-generation sequencing
PCR: Polymerase chain reaction
RdRp: RNA-dependent RNA polymerase
RoAstVs: Rodent AstVs
RoBAstVs: Rodent BAstVs
